# Effects of Acute Hypoxia and Reoxygenation on Physiological and Immune Responses and Redox Balance of Wuchang Bream (*Megalobrama amblycephala* Yih, 1955)

**DOI:** 10.3389/fphys.2017.00375

**Published:** 2017-06-08

**Authors:** Nan Chen, Meng Wu, Guo-Pan Tang, Hui-Juan Wang, Chun-Xiao Huang, Xin-Jie Wu, Yan He, Bao Zhang, Cui-Hong Huang, Hong Liu, Wei-Min Wang, Huan-Ling Wang

**Affiliations:** ^1^Key Lab of Freshwater Animal Breeding, Key Laboratory of Agricultural Animal Genetics, Breeding and Reproduction, Ministry of Education, College of Fishery, Huazhong Agricultural UniversityWuhan, China; ^2^Freshwater Aquaculture Collaborative Innovation Center of Hubei ProvinceWuhan, China; ^3^Laboratory of Freshwater Animal Breeding, College of Animal Science and Technology, Henan University of Animal Husbandry and EconomyZhengzhou, China

**Keywords:** *Megalobrama amblycephala*, hypoxia, physiological response, immune activity, oxidative stress

## Abstract

To study *Megalobrama amblycephala* adaption to water hypoxia, the changes in physiological levels, innate immune responses, redox balance of *M*.amblycephala during hypoxia were investigated in the present study. When *M. amblycephala* were exposed to different dissolved oxygen (DO) including control (DO: 5.5 mg/L) and acute hypoxia (DO: 3.5 and 1.0 mg/L, respectively), hemoglobin (Hb), methemoglobin (MetHb), glucose, Na^+^, succinatedehydrogenase (SDH), lactate, interferon alpha (IFNα), and lysozyme (LYZ), except hepatic glycogen and albumin gradually increased with the decrease of DO level. When *M. amblycephala* were exposed to different hypoxia time including 0.5 and 6 h (DO: 3.5 mg/L), and then reoxygenation for 24 h after 6 h hypoxia, Hb, MetHb, glucose, lactate, and IFNα, except Na^+^, SDH, hepatic glycogen, albumin, and LYZ increased with the extension of hypoxia time, while the above investigated indexes (except albumin, IFNα, and LYZ) decreased after reoxygenation. On the other hand, the liver SOD, CAT, hydrogen peroxide (H_2_O_2_), and total ROS were all remained at lower levels under hypoxia stress. Finally, Hif-1α protein in the liver, spleen, and gill were increased with the decrease of oxygen concentration and prolongation of hypoxia time. Interestingly, one Hsp70 isoforms mediated by internal ribozyme entry site (IRES) named junior Hsp70 was only detected in liver, spleen and gill. Taken together, these results suggest that hypoxia affects *M. amblycephala* physiology and reduces liver oxidative stress. Hypoxia-reoxygenation stimulates *M. amblycephala* immune parameter expressions, while Hsp70 response to hypoxia is tissue-specific.

## Introduction

Oxygen is critical for both terrestrial and aquatic aerobic organisms. Most of the organisms need molecular oxygen to support metabolic processes (Chandel and Schumacker, 2000). Compared with terrestrial environments, aquatic environments exhibit much wider temporal and spatial variations in oxygen concentration, and water contains extremely lower oxygen in the same volume of air at the same atmosphere pressure (Rytkönen et al., 2007). As a result, aquatic organisms are frequently exposed to variations in oxygen levels. In most cases, they will usually respond by escaping to other environments when encountering hypoxia condition. However, if hypoxia is unavoidable, aquatic organisms including fish have to make responses either by increasing oxygen transfer to tissues or reducing oxygen consumption through metabolic depression to survive (Moraes et al., 2002; Douxfils et al., 2012).

The earlier studies of fish under hypoxia stress are focused on tissue changes including blood, heart, gill, and brain. In rainbow trout, hypoxia markedly decreases the oxygen uptake effectiveness in blood (Randall et al., 1967), whereas during progressive hypoxia, the rate of oxygen uptake does not alter (Holeton and Randall, 1967b). During hypoxia the percentage saturation of both arterial and venous blood are decreased, while blood pressure in the dorsal and ventral aortae increases, all these changes are associated with a marked bradycardia in fish after hypoxia stress (Holeton and Randall, 1967a). Bradycardia does not affect fish cardiac output but stroke volume of cardiac enhanced, and vascular resistance to blood flow correspondingly increased (Holeton and Randall, 1967b; Butler and Taylor, 1975). Hypoxia also increases fish Hb-O_2_ affinity and oxygen capacity, and directly provides higher and more stable effectiveness of oxygen uptake in blood; however the total *P*_*O*2_ is till decreased (Holeton and Randall, 1967b; Wood and Johansen, 1972). During hypoxia, the ATP concentration and ATP:Hb ratio in the red cells decreased (Wood et al., 1975), further demonstrate that the erythrocytic ATP:Hb4 molar ratios declined with increasing hypoxic stress as did the pH gradient between the erythrocyte and plasma (Boutilier et al., 1988). Hypoxia leads to the fish bradycardia, while the cardiac output is regulated by changes in the stroke volume not heart rate (Hemmingsen et al., 1972). Long period of hypoxia has no effect on fish heart functions but leads to the decrease of myocardial glycogen concentration and the occurrence of mitochondrial necrosis (Leknes, 1985; Lennard and Huddart, 1992). Primarily, fish moves higher in the water column, increases gill ventilation and exhibits aquatic surface respiration during aquatic hypoxia (Urbina et al., 2011). Gills display protruding lamellae after 1 day of hypoxia and reach its greatest extent after 7 days, and the changes are completely reversible after 7 days reoxygenation (Sollid et al., 2003). The capillary diameter of fish brain increases after hypoxia, and 90 min hypoxia leads to the disappearance of brain pyruvate kinase (Scheich et al., 1972; Lushchak, 1993). Brain sensitivity to hypoxia in fish is reflected by changes in extracellular K^+^ activity, and in the hypoxia-tolerant fish crucian carp the Na^+^/K^+^-ATPase level in hypoxia crucian carp brain is maintained (Hylland et al., 1997).

The recent studies show that some hematological parameters including red and white blood cell count, hematocrit and hemoglobin concentrations are significantly increased after hypoxia treatment in fish (Petersen and Gamperl, 2011; Richards, 2011; Ni et al., 2014). Meanwhile, the biochemical parameters, such as blood glucose, lactate, corticosteroids, ATP, and GTP are also altered under hypoxia (Rees et al., 2009; Omlin and Weber, 2010; Urbina and Glover, 2012; Ni et al., 2014). During hypoxia, metabolic activity decreases and most of the energy will be used for the maintenance of primary physiological functions, probably leading to the changes of the immune defense. It is reported that in Eurasian perch and Catla, the lysozyme activity is significantly decreased under hypoxia conditions (Douxfils et al., 2012; Singh et al., 2016).

During the aerobic metabolism, the negative tetravalent atom of O_2_ produces water at the end of the mitochondrial electron transport chain, while negative monovalent atom of O_2_ generates several reactive oxygen species (ROS) such as superoxide anions (${\text{O}}_{2}^{-•}$), hydroxyl anions (OH^−•^), and hydrogen peroxide (H_2_O_2_) (Turrens, 2003). These ROS have damage effect on organisms including induction of oxidation of proteins, DNA and steroid components, and peroxidation of unsaturated lipids in cell membranes (Chance et al., 1979; Southorn and Powis, 1988). To minimize the detrimental influence of ROS, aerobic organisms have evolved enzymatic antioxidant mechanism accompanying the formation of antioxidant defenses such as superoxide dismutases (SOD), catalases (CAT), and glutathione (GSH), to scavenge the superoxide radicals and hydrogen peroxide for maintaining the homeostasis (Scandalios, 2005; Kobayashi and Yamamoto, 2006). SOD catalyzes the dismutation of ${\text{O}}_{2}^{-•}$ to H_2_O_2_, and CAT and glutathione peroxidases (GSH-Px) reduce H_2_O_2_ to H_2_O (Scandalios, 2005). When the ROS generation exceeds their removal, oxidative stress occurs. It has been reported that hyperoxia or even hypoxia alters cellular ROS levels in fish (Ross et al., 2001; Cooper et al., 2002; Garcia Sampaio et al., 2008). In some fish species, hypoxia alone causes elevation of the activities of enzymes involved in antioxidant defense (Lushchak et al., 2001, 2005; Lushchak and Bagnyukova, 2007), while in other fish species the antioxidant enzyme activities are decreased or/and unchanged in hypoxia stress (Leveelahti et al., 2014; Huang et al., 2015; Ransberry et al., 2016). Moreover, ROS-detoxifying enzyme activities rather than direct reactive intermediate levels are investigated in most experiments to indicate the altered status of ROS in hypoxia.

*Megalobrama amblycephala* (Wuchang bream) as an important freshwater fish has been cultured for half a century, whereas intensive aquaculture has brought lots of problems, such as crowding and hypoxia. More seriously, the absence of oxygen in water usually causes the large-scale suffocation death of *M. amblycephala*, thus resulting in great economic losses. In the present study, *M. amblycephala* were treated by different dissolved oxygen (DO) concentration and hypoxia time to systematically study their responses to hypoxia in physiological and immune levels by evaluating blood parameters. In addition, oxidative stress status in hypoxia was studied by analyzing ROS and related antioxidant enzymes in liver. Finally, the distributions of the stress response proteins in different tissues were also investigated.

## Materials and methods

### Experimental fish

Juvenile *M. amblycephala* (mean weight 50 ± 10 g) were obtained from Tuanfeng breeding base in Hubei province, China. The fish were acclimated for 2 weeks with daily diet under a 14/10 h day/night photoperiod cycle at 25°C in one 500 L tank with circulating water system.

### Hypoxia stress

The DO concentration was maintained by flowing the nitrogen gas into the 100 L hermetic tank by a self-designed automatic hypoxia control machine. The DO concentration was set based on the previous studies (Wang et al., 2015). Fish were randomly divided into DO level groups (*n* = 5 for each group): the control (CTRL-5.5, DO: 5.5 ± 0.2 mg/L), acute hypoxia 1 group (aH1-3.5, DO: 3.5 ± 0.2 mg/L for 0.5 h), and acute hypoxia 2 group (aH2-1.0, DO: 1.0 ± 0.2 mg/L for 0.5 h); hypoxia time groups: comparable-acute hypoxia group (cH-0.5h, DO: 3.5 ± 0.2 mg/L for 0.5h), comparable-acute hypoxia 1 group (cH1-6h, DO: 3.5 ± 0.2 mg/L for 6 h), and reoxygenation group (rH-24h, DO: 3.5 mg/L for 6 h and then recovered to DO: 5.5 mg/L for 24 h). The aH1-3.5 (from DO level groups) and cH-0.5h (from hypoxia time groups) were treated with same hypoxia conditions and the samples were shared. Fish were immediately anesthetized with MS-222 (150 mg/L), and sampled at the end of each experimental period when fish gill ventilation ceased. Fish were not fed during the experiment.

All investigations were conducted in accordance with the ethical standards and according to the national and international guidelines, and this study has been approved by the Institutional Animal Care and Use Committee (IACUC) of Huazhong Agricultural University, Wuhan, China.

### Blood and tissue sampling

Blood samples were taken gently from the caudal vein by 1 mL plastic syringe without heparin sodium. These samples were incubated at 37°C for 2 h and clotted at 4°C, then centrifuged at 4,000 g for 10 min to obtain the serum.

Tissues including liver, spleen, brain, gill and kidney were excised and subsequently frozen in liquid nitrogen. The frozen samples were then thawed and homogenized individually using TissueLyser ∏ (Qiagen, Germany) at the frequency of 30 Hz for 5 min in ice-cold RIPA lysis buffer at a ratio of 1:10 (Cowin Biotech, China). The homogenates were further centrifuged at 12,000 g for 10 min at 4°C, and the supernatant was used for western blot analysis. Liver was also homogenized in 0.65% (m/V) brine at a ratio of 1:10, and after centrifugation, the supernatant was used for enzyme activity assays. All the protein concentrations were determined by bicinchoninic acid (BCA) protein determination kit (Dingguo, China) according to the instructions.

### Hematological and biochemical assays

Hemoglobin (Hb) concentration was determined with Drabkin's reagent as an absorbance at 540 nm. MetHb was computed from Hb by double-wavelength spectrophotometry method (Sakata et al., 1982). Biochemical parameters including Na^+^, glucose, lactate, and hepatic glycogen were determined by commercial kits according to chromogenic ionophore-based method (Kumar et al., 1988), glucose oxidase/peroxidase method (Trinder, 1969), p-phenilphenol method (Harrower and Brown, 1972), and modified glycogen method (Bidinotto et al., 1997), respectively.

### ROS and antioxidant defense assays in liver

An oxidant-sensing fluorescent probe 2′,7′-dichlorodihydrofluorescein diacetate (DCFH-DA) was used to determine the total status of ROS as previously described (Li et al., 1999; Cash et al., 2007). Other two reactive intermediates, H_2_O_2_ and nitric oxide (NO) were determined by phenol red (Pick and Mizel, 1981) and hemoglobin methods (Murphy and Noack, 1994), respectively. The SOD activity was measured based on the ability of the enzyme to inhibit the reduction of nitro blue tetrazolium (NBT) by superoxide radicals (Crouch et al., 1981). The CAT activity was measured using the catalase assay kit (Jiancheng, China).

### Immune parameter detection

Serum albumin was evaluated following the bromcresol green (BCG) method (Barber and Stanhope, 1992). Serum interferon alpha (IFNα) protein level was studied with the ELISA kit (Jiancheng, China). Serum lysozyme (LYZ) activity in mixing serum sample (20 μL) with 200 μL *Micrococcus luteus* solution was measured for transmittance at 530 nm after 15 min incubation at 37°C, and assessed by comparing transmittance values with reference standards.

### Western blot analysis

Total proteins from *M. amblycephala* liver, spleen, brain, gill, and kidney (*n* = 5 for each group) were quantified and separated on 8% SDS-PAGE, then transferred to nitrocellulose membranes. The membranes were incubated for 2 h with polyclonal antibodies, Hif-1α (1:500) and Hsp70 (1:200) (Boster, China), respectively, and then anti-rabbit IRDye 800CW-labeled secondary antibody (1:10,000) at room temperature for 1 h, and observed using Odyssey Fc machine (Licor Biosciences, USA). Gray values of every band were further calibrated and measured by ImageJ 1.46r (NIH, USA).

### Statistics

Statistical analysis was performed using SPSS 16.0 software. Data from each groups were, respectively, analyzed by the one-way ANOVA test followed by the Duncan's new multiple range tests. Data were represented as mean ± *S.D*. and *p* < 0.05 was considered statistically significant.

## Results

### Hypoxia significantly increased hematological and biochemical parameters

**Figure 1:**
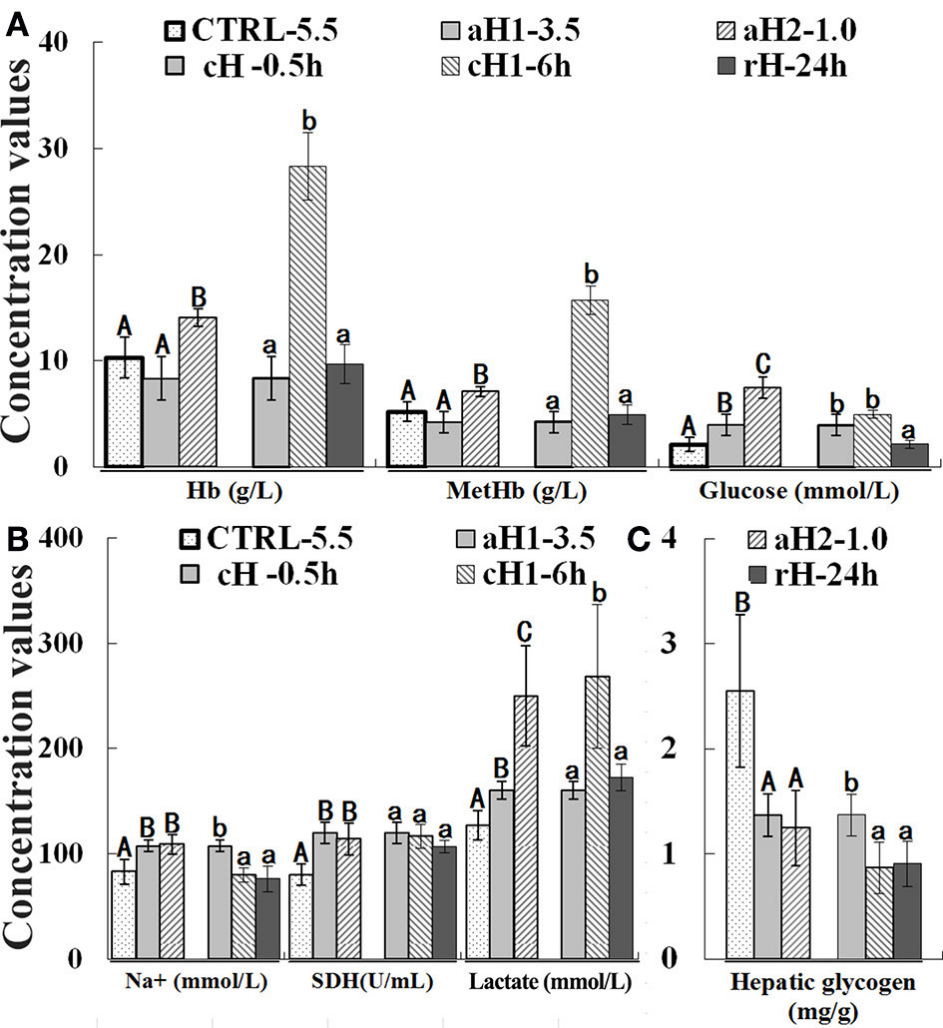
Effect of hypoxia upon hemoglobin (Hb), methemoglobin (MetHb), and glucose **(A)**, Na^+^, succinatedehydrogenase (SDH) and lactate **(B)**, and hepatic glycogen **(C)** concentrations in *Megalobrama amblycephala*. CTRL-5.5, the control; aH1-3.5 and aH2-1.0, acute hypoxia at 1.0 and 3.5 mg/L of DO last for 0.5 h, respectively; cH-0.5h and cH1-6h, comparable-acute hypoxia (DO: 3.5 mg/L) last for 0.5 and 6 h, respectively; rH-24h, hypoxia (DO: 3.5 mg/L) last for 6 h and then recover to DO: 5.5 mg/L for 24 h. Three different DO level groups are individually analyzed and indicated with capital letters, while three hypoxia time condition groups are also separately analyzed and showed with lowercase letters. Different letters above bars represent significant difference (*p* < 0.05), and the same letters above bars indicate no significant difference. Values are mean ± S.D.; *N* = 5 for every groups.

Compared with the control, the Hb and MetHb concentrations increased with the decrease of oxygen concentration and the prolongation of hypoxia time, however, after 24 h reoxygenation, their concentrations returned to the normal levels (Figure 1A). Glucose contents increased in an oxygen concentration-, but not hypoxia time- dependent manner, and recovered to the normal levels after reoxygenation (Figure 1A).

Na^+^ concentration gradually increased with the decrease of DO concentration, whereas it decreased with the prolongation of hypoxia time, and recovered to the normal levels after reoxygenation (Figure 1B). SDH activity showed the same tendency with Na^+^ during hypoxia treatment. Additionally, with the decrease of DO and prolongation of hypoxia time, lactate concentration increased and reached the maximum (268.43 mmol/L) at moderate hypoxia (DO: 3.5 mg/L) treatment for 6 h (Figure 1B). On the other hand, the hepatic glycogen concentration was continuously decreased during hypoxia and reoxygenation (Figure 1C).

### Hypoxia stimulated *M. amblycephala* immune activity

**Figure 2:**
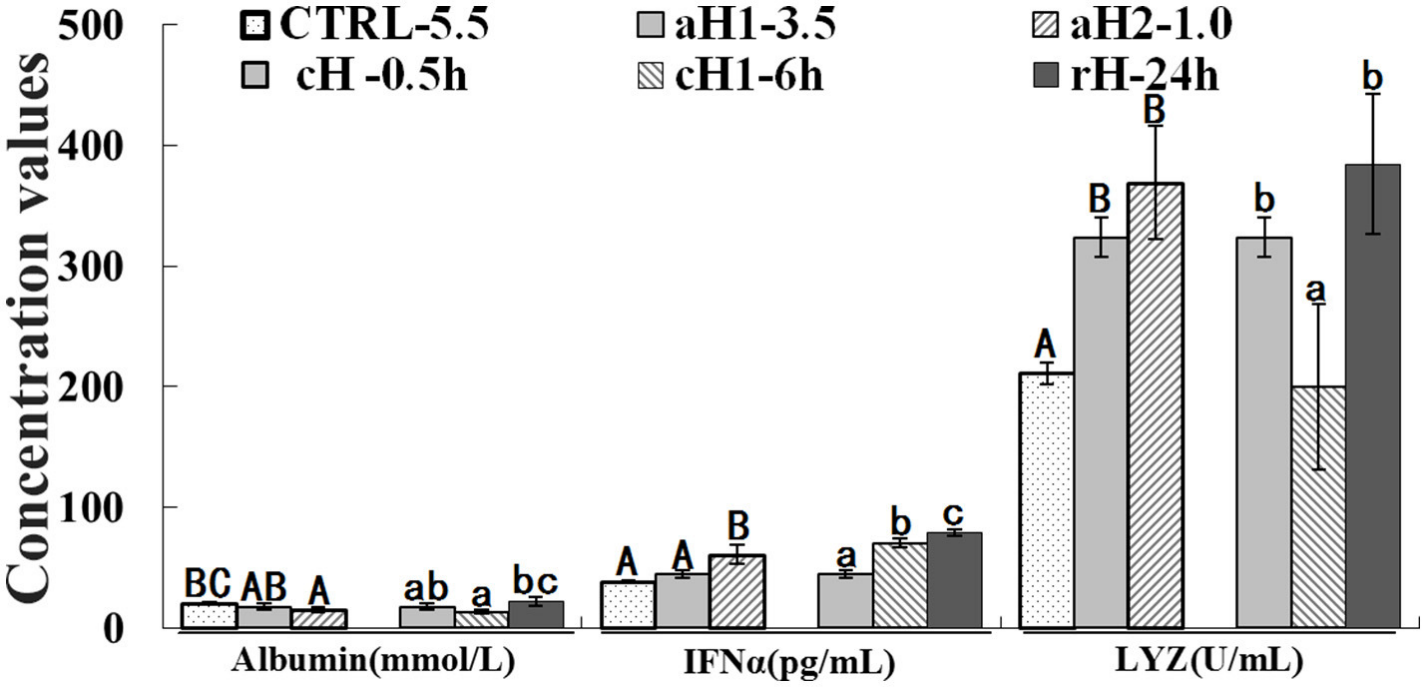
Serum albumin, interferon alpha (IFNα) and lysozyme (LYZ) levels in *M. amblycephala* after hypoxia treatment. Three different DO level groups are individually analyzed and indicated with capital letters, while three hypoxia time condition groups are also separately analyzed and showed with lowercase letters. Different letters above bars represent significant difference (*p* < 0.05), and the same letters above bars indicate no significant difference. Values are mean ± S.D.; *N* = 5 for every groups.

The albumin concentration declined in hypoxia, but returned to the normal level after reoxygenation. Conversely, the IFNα content showed an increase tendency with the decrease of DO concentration and prolongation of hypoxia time. The LYZ level significantly increased after hypoxia treatment. It should be emphasized that the albumin, IFNα, and LYZ concentrations were stayed at higher levels after hypoxia-reoxygenation compared to the control (~2.1- and 1.8-fold increase for IFNα and LYZ, respectively; Figure 2).

### Hypoxia generally decreased antioxidant enzyme activities and ROS production

**Figure 3:**
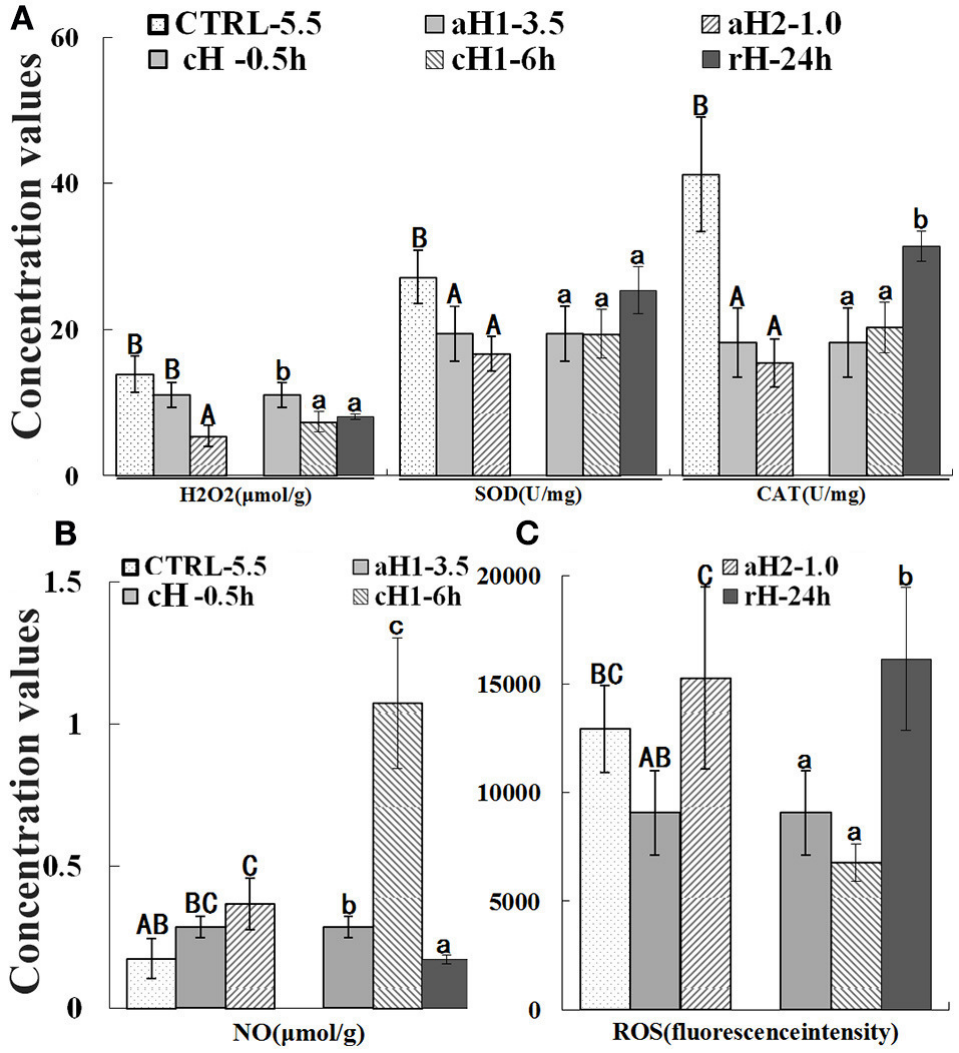
Hydrogen peroxide (H_2_O_2_) concentration, superoxide dismutase (SOD), and catalases (CAT) activity **(A)**, nitric oxide (NO) concentration **(B)**, and total reactive oxygen species (ROS) **(C)** in *M. amblycephala* liver after hypoxia treatment. Three different DO level groups are individually analyzed and indicated with capital letters, while three hypoxia time condition groups are also separately analyzed and showed with lowercase letters. Different letters above bars represent significant difference (*p* < 0.05), and the same letters above bars indicate no significant difference. Values are mean ± S.D.; *N* = 5 for every groups.

The activities of antioxidant enzymes, SOD, and CAT were detected in the liver, and the results indicated that both SOD and CAT activities were declined with the decrease of DO concentration and reached the minimum level (16.73 and 15.46 U/mg, respectively) under severe acute hypoxia (DO: 1.0 mg/L) condition (Figure 3A). Interestingly, the concentration of H_2_O_2_, the CAT substrate, also showed the similar change tendency with the SOD and CAT activities. After hypoxia-reoxygenation, SOD and CAT activities increased and were comparable to the control (*T-*test: *p*-values for SOD and CAT are 0.272 and 0.052, respectively). The NO content increased with the decrease of DO and prolongation of hypoxia time (Figure 3B). The total ROS concentration fluctuated under hypoxia, which decreased at 0.5 h hypoxia (DO: 3.5 mg/L) treatment, but significantly increased when DO was continually dropped to 1.0 mg/L (Figure 3C). As shown in Figure 3, these detected reactive species H_2_O_2_ and ROS were all retained a lower level in hypoxia but a higher level in normoxia or reoxygenation.

### Hypoxia response in *M. amblycephala* was tissue-specific

**Figure 4:**
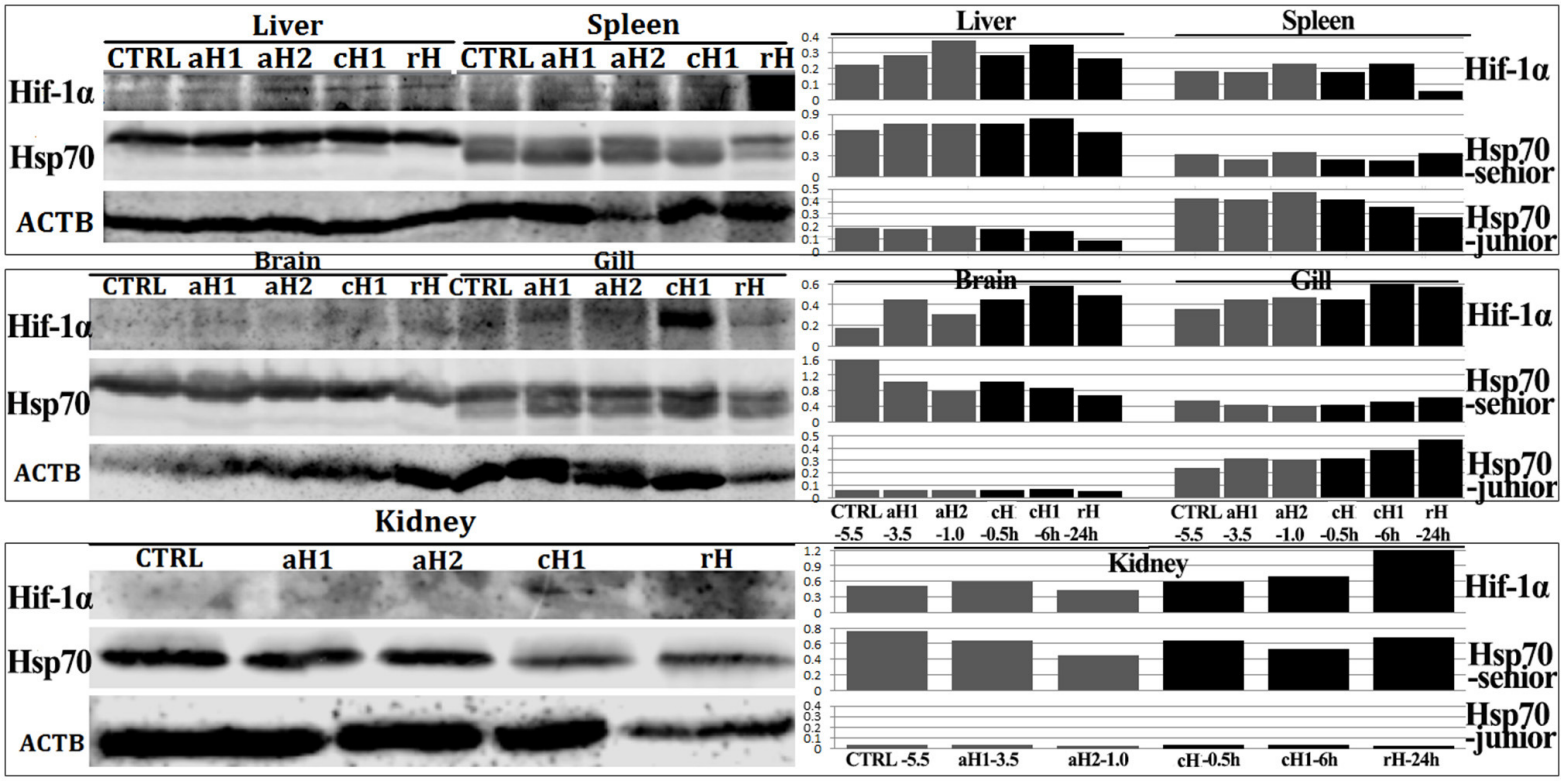
Western blot analysis of Hif-1α and Hsp70 proteins in different tissues after hypoxia treatment in *M. amblycephala*. CTRL-5.5, the control; aH1-3.5 and aH2-1.0, acute hypoxia at 1.0 and 3.5 mg/L of DO last for 0.5 h, respectively; cH-0.5h and cH1-6h, comparable-acute hypoxia (DO: 3.5 mg/L) last for 0.5 and 6 h, respectively; rH-24h, hypoxia (DO: 3.5 mg/L) last for 6 h and then recover to DO: 5.5 mg/L for 24 h. The lower band in liver, spleen and gill is the junior Hsp70 protein generated by IRES-mediated translation. The left sides are western blot result, while the right sides are corresponding ratio of gray values of Hif-1α and Hsp70 proteins.

The key hypoxia response factor, Hif-1α protein was induced by hypoxia in liver, spleen, brain, gill and kidney (Figure 4). Hif-1α levels were gradually increased in oxygen concentration- and hypoxia time-dependent manner in liver, spleen, and gill, while in brain and kidney, Hif-1α was firstly increased and then decreased. Meanwhile, the more accumulation of Hif-1α protein was observed with the prolongation of hypoxia time in liver, spleen, brain, and especially in gill. However, after 24 h hypoxia-reoxygenation, Hif-1α was decreased (Figure 4).

The two Hsp70 isoforms including the junior one generated by IRES were detected in liver, spleen, brain, gill, and kidney of *M. amblycephala*. The two isoforms in liver had similar expression tendency which remained at higher levels during hypoxia treatment and decreased after hypoxia-reoxygenation. In spleen and gill, the senior Hsp70 had higher levels in normoxia or hypoxia-reoxygenation, while the junior one increased after hypoxia treatment. In brain and kidney, the junior Hsp70 was undetectable, but the senior one responded to hypoxia in oxygen concentration- and time-dependent manner (Figure 4).

## Discussion

In order to provide insight into mechanisms of the hypoxia adaptation in physiology, immune response and oxidative stress in *M. amblycephala*, some well-known hypoxia indicators, Hb, MetHb, glucose, lactate, glucose, and hepatic glycogen concentrations were investigated after hypoxia stress, and showed the consistency with the previous studies, for instance, Hb and MetHb, functioning in oxygen transport and utilization are induced when the pressure of oxygen decreases in blood (Grek et al., 2011). Our results show that Hb and MetHb are more significantly induced at 6 h for moderate hypoxia treatment, suggesting that Hb and MetHb may be more sensitive to hypoxia time rather than oxygen levels in fish. Na^+^ influx in fish erythrocytes can indicate the activation of Na^+^/H^+^ exchanger and the increase of the pH value, inducing a decrease of hemoglobin affinity to oxygen in fish erythrocytes, as a result the oxygen is delivered into tissues (Nikinmaa, 1992; Reid and Perry, 1994; Nielsen, 1997). In this study, Na^+^ stayed at high levels with the decrease of DO, but decreased at 6 h for hypoxia, indicating that Na^+^ probably flows into *M. amblycephala* erythrocytes (Figure 1). SDH is involved in both the citric acid cycle and respiratory electron transfer chain (Rutter et al., 2010), and its activity under hypoxia stress was obviously increased in *M. amblycephala*.

During the hypoxia treatment, glucose increased while the hepatic glycogen content decreased, which has also been reported in fish and other species (Routley et al., 2002; Douxfils et al., 2012; Polak et al., 2013). It is suspected that hepatic glycogen is the primary source of glucose, when animals are subjected to acute hypoxia, glycogenolysis is enhanced and hepatic glucose is released to blood to provide sufficient blood glucose and energy (through anaerobic glycolysis), thus ensure adequate basal metabolism for organism survival (Chen et al., 2007). Therefore, glucose homeostasis during the hypoxia treatment depends primarily on hepatic glucose output (Wahren and Ekberg, 2007).

It is well-known that the ROS increase naturally stimulates the activities of antioxidant enzymes, SOD, CAT, GSH-Px, GST, etc. to remove ROS (Niki, 2010). However, the increase of antioxidant enzyme activities does not mean the ROS generation, or vice versa, especially under combined-factor conditions. Additionally, the antioxidant enzyme levels could only reflect the antioxidant capacity rather than the status of oxidative stress. Thus, the levels of the reactive intermediates, H_2_O_2_ and ROS are analyzed and the results showed that hypoxia decline H_2_O_2_ and ROS levels integrally. However, ROS reaches the peak after severe acute hypoxia (DO: 1.0 ± 0.2 mg/L) treatment for 0.5 h, suggesting that anaerobic metabolism has probably taken the place of aerobic metabolism during this DO point since *M. amblycephala* suffocation point ranges from 0.64 to 0.35 mg/L (Chen et al., 2012). Meanwhile, the lactate level representing anaerobic metabolism also reached the peak, indicating the anaerobic metabolism is occurred (Segura et al., 1996). The activities of the antioxidant enzymes (SOD, CAT), and the H_2_O_2_ and ROS contents retained at lower levels under hypoxia conditions, which are consistent with some published studies in fish, but contrary with others (Table S1). After careful and scrupulous analysis and comparison, we suggest that the biomarkers of oxidative stress caused by hypoxia and especially co-hypoxia factors probably be primarily focused on ROS itself. Moreover, moderate hypoxia treatment may positively affect *M. amblycephala* by driving liver into lower oxidative stress status. It is reported that chronic hypoxia leads to a marked improvement in mouse survival, body weight, body temperature, behavior, and enhancement against mitochondrial toxicity (Jain et al., 2016).

It has been known that some eukaryotic mRNAs can be translated via internal initiation by specific mRNA regions termed internal ribosome entry site (IRES) (Holcik et al., 1999; Cornelis et al., 2000). Remarkably, many IRES-containing mRNAs encode proteins that have important roles in development, differentiation, cell cycle progression, cell growth, cell apoptosis, and stress response (Holcik et al., 1999; Henis-Korenblit et al., 2000; Stoneley et al., 2000; Prats and Prats, 2002). The presence of IRES elements allows crucial survival factors to be transiently translated under stress conditions that require immediate changes in protein levels (Hellen and Sarnow, 2001; Holcik and Sonenberg, 2005; Komar and Hatzoglou, 2005). The IRES on 5′-UTR of *Hsp70* has been studied to drive cap-independent Hsp70 protein translation in apoptosis, hypertonic and thermal stress (Rubtsova et al., 2003; Hernández et al., 2004; Rocchi et al., 2013; Chen et al., 2014). Our results firstly indicate that Hsp70 could respond to hypoxia stress through IRES-mediated manner, moreover, the Hif-1α and junior Hsp70 expressions in limited tissues indicate that hypoxia stress induce tissue-specific response.

Taken together, we have performed hypoxia/reoxygenation treatment in *M. amblycephala* and systematically studied their responses to hypoxia in aspect of physiology, oxidative stress, and immune. The results demonstrate that hypoxia significantly induces *M. amblycephala* hematological and biochemical parameters, and moderate hypoxia drives the liver into a lower oxidative stress status to protect fish from oxidative damage. Furthermore, we have firstly observed that hypoxia-reoxygenation treatment stimulates *M. amblycephala* innate immune activity.

## Author contributions

NC and HLW conceived and designed the experiments. MW, HJW, CXH, XW, YH, BZ, and CHH carried out the experiments and analyzed the data. NC, WW, HL, and HLW supervised the project. NC wrote the manuscript and all authors reviewed the manuscript.

### Conflict of interest statement

The authors declare that the research was conducted in the absence of any commercial or financial relationships that could be construed as a potential conflict of interest.
