# Supplementary material for: Effects of Acute Hypoxia and Reoxygenation on Physiological and Immune Responses and Redox Balance of Wuchang Bream (Megalobrama amblycephala Yih, 1955)
Source: Front Physiol. 2017 Jun 8;8:375. doi: 10.3389/fphys.2017.00375 (PMC5462904; doi:10.3389/fphys.2017.00375)
Supplement: Supplementary file 1 [file Table1.DOC]

Table S1 Collections of studies with fish exposed to several hypoxia/reoxygenation conditions and these fish liver are sampled for measurement of redox stress parameters and antioxidase enzyme activity.

| Study and time | Latin name | Fish name | Hypoxia/anoxia conditions | ROS related indicators in liver (↑ and ↓ mean up- and down-regulation after reoxygenation, respectively) | | | Antioxidase related indicators in liver (↑ and ↓ mean up- and down-regulation after reoxygenation, respectively) | | |
| --- | --- | --- | --- | --- | --- | --- | --- | --- | --- |
|  |  |  |  | Up regulated | Unchanged | Down regulated | Up regulated | Unchanged | Down regulated |
| Ransberry et al., 2016 | *Fundulus heteroclitus* | Killifish | DO: 9.1; 2.33, 2.51; 96 h | - | TBARS | - | - | SOD | CAT, |
| Du et al., 2016 | *Fundulus heteroclitus* | Killifish | DO: 2.0; 28 d  DO: 8.0; 28 d | - | - | H_2_O_2_ | SOD, CAT | - | - |
| Zhang et al., 2016 | *Pelteobagrus vachelli* | Darkbarbel catfish | DO: 6.8, 0.7; 1.5, 4, 6.5 h | LP(↓), MDA(↓) | - | - | SOD(↓), | - | - |
| Mustafa et al., 2015 | *Cyprinus carpio* | Mirror carp | DO: 1.8; 21 d  DO: 8.2; 7 d | ODD(↑), TBARS(↓) | - | - | - | - | - |
| Huang et al., 2015 | *Trichogaster microlepis* | Moonlight gourami | DO: 1.0; 3, 12 h  DO: 7.9; 12 h | - | - | - | - | SOD(↓), CAT,  GPx | - |
| Leveelahti et al., 2014 | *Gasterosteus aculeatus* | Threespine sticklebacks | DO: 24%; 3 h | - | - | - | - | SOD, CAT, GPx, GST, G6PDH | - |
| Leveelahti et al., 2014 | *Oncorhynchus mykiss* | Rainbow trout | DO: 33%; 48 h | - | - | - | - | SOD, CAT, GST, G6PDH | - |
| Hauser-Davis et al., 2014 | *Piaractus mesopotamicus* | Pacu specimens | DO: 0.5; 24 h | - | TBARS | - | - | - | GSH, MT |
| Okogwu et al., 2014 | *Carassius auratus* | Goldﬁsh | DO: 0.8; 48 h  DO: 7.0; 48 h | - | - | - | SOD(↑) | TAOC,CAT, GPx(↓) | - |
| Dolci et al., 2014 | *Rhamdia*  *quelen* | Silver catfish | DO: 2.8; 10 d | - | RS | TBARS | - | - | CAT |
| Tripathi et al., 2013 | *Clarias batrachus* | Indian catfish | DO: 0.98; 1, 2, 3, 6, 12 h | - | - | - | - | GSH, CAT | SOD |
| Welker et al., 2012 | *Oreochromis niloticus* | Nile tilapias | DO: 0.28; 3 h  DO: 7.0; 0.5, 2, 6, 12, 24 h | - | TBARS | - | - | CAT, GPx, SOD(↑), GST | GSH(↑) |
| Pérez-Jiménez et al., 2012 | *Sparus aurata* | Gilthead sea  bream | DO: 2.8; 3, 6 h  DO: 7.1; 3, 6 h | LP(↓) | - | OSI(↑) | GPx(↓) | G6PDH, CAT | SOD(↑), GSH(↑), GSSG(↑) |
| Riffel et al., 2012 | *Leporinus macrocephalus* | Piavucu | DO: 6.1, 4.0, 3.2, 2.5, 0.71; 96 h | - | - | TBARS, LHP | - | - | SOD, CAT, GST |
| Kubrak et al., 2012 | *Carassius auratus* | Goldfish | Mimic hypoxia: 10, 25, 50; 96 h | - | - | GR | CAT | SOD, GPx, G6PDH | GST |
| Keleştemur, 2012 | *Oncorhynchus mykiss* | Rainbow trout | DO: 7.0, 4.5, 3.5; 8 h | MDA | - | - | - | - | - |
| Azambuja et al., 2011 | *Rhamdia*  *quelen* | Silver catfish | DO: 13.3, 7.4, 2.3; 5, 6, 7 h | - | - | TBARS | - | - | SOD, CAT, GST |
| Mustafa et al., 2011 | *Cyprinus carpio L* | Common carp | DO: 7.1, 1.8, 12.3; 30 d | ODD(↑) | - | - | - | - | GPx |
| Garcia et al., 2008 | *Cyprinus carpio* L | Common carp | DO: 50 mm Hg; 48 h | - | LHP | - | - | SOD | CAT, GPx |
| Lushchak and Bagnyukova, 2007 | *Perccottus glenii* | Chinese sleeper | DO: 0.4; 2, 6, 10 h  DO: 6.5; 1, 24 h | - | - | TBARS | SOD | GST, G6PDH | CAT(↑), GPx(↑), GR(↑), GR(↑) |
| Heise et al., 2006 | *Zoarces viviparus* | North Sea eelpout | Mimic hypoxia: | - | TBARS(↑) | - | GSH(↓) | - | SOD, GPx, GR(↑), GSSG(↑) |
| Olsvik et al., 2006 | *Gadus morhua* | Atlantic cod | DO: 46.2%; 6 w  DO: 76.4%; 6 w  DO: 145.2%; 6 w | - | - | - | - | - | SOD(↑), GPx(↑) and CAT(↑) mRNA |
| Lushchak et al., 2005 | *Cyprinus carpio* L | Common carp | DO: 0.9; 5h  DO: 3.6; 1, 14 h | TBARS | - | LP | GSH(↑) | CAT, GPx, GR | SOD(↑) |
| Wilhelm Filho et al., 2005 | *Leporinus elongatus* | Piapara | DO: 1.9; 14 d  DO: 3.9; 7 d  DO: 6.9; 7 d | - | - | TBARS(↑) | GSSG, GPx(↓), GST | GSH, CAT, GR | - |
| Cooper et al., 2002 | *Leiostomus xanthurus* | Estuarine | DO: 0.8, 2.0, 4.0, 8.0; 12 h | - | - | - | - | SOD | CAT(↑) |
| Lushchak et al., 2001 | *Carassius auratus* | Goldfish | Hypoxia: 8 h  Normal: 1, 14 h | - | - | - | SOD, CAT(↑), GST, G6PDH(↓) | - | GPx(↑), GR(↑) |
| Lind, 1992 | *Carassius carassius* | Crucian carp | Normal: June  Hypoxia: Sept. | - | - | - | - | cyt-ox | - |
| Radi et al., 1988 | *Cyprinus carpio L* | Common carp | Hyperoxia  Hypoxia  Anoxia | LP | - | - | GPx | - | SOD |
| Cann-Moisan et al., 1988 | *Oncorhynchus mykiss* | Rainbow trout | Hypoxia: 101 ATA | - | - | - | - | - | ATP |
| Arillo et al., 1984 | *Oncorhynchus mykiss* | Rainbow trout | Hypoxia | - | - | - | - | - | ATP |
| Burton and Spehar, 1971 | *Oncorhynchus mykiss* | Rainbow trout | DO: 1.0; 15-50 min  DO:11.9; 15-50 min  DO: 9.4, 8.6, 4.2, 1.5, 0.5, 0; 4.5-8 h | - | - | - | - | Free SCFAs | - |
| Burton and Spehar, 1971 | *Salmo trutta* | Brown trout | DO: 1.0; 15-50 min  DO:11.9; 15-50 min  DO: 9.4, 8.6, 4.2, 1.5, 0.5, 0; 4.5-8 h | - | - | - | - | Free SCFAs | - |
| Burton and Spehar, 1971 | *Lepomis macrochirus* | Bluegill sunfish | DO: 1.0; 15-50 min  DO:11.9; 15-50 min  DO: 9.4, 8.6, 4.2, 1.5, 0.5, 0; 4.5-8 h | - | - | - | - | Free SCFAs | - |
| Burton and Spehar, 1971 | *Ictalurus nebulosus* | Brown bullhead catfish | DO: 1.0; 15-50 min  DO:11.9; 15-50 min  DO: 9.4, 8.6, 4.2, 1.5, 0.5, 0; 4.5-8 h | - | - | - | - | Free SCFAs | - |

Abbreviations: DO, dissolved oxygen (mg/L) or percentage of air saturation; h, hours; d, days; w, weeks; ODD, oxidative damage to DNA; TBARS, thiobarbituric acid reactive substance (lipid injury); G6PDH, glucose 6-phosphate dehydrogenase; GPx, glutathione peroxidase; MT, metallothioneins; RS, reactive species; TAOC, total antioxidant capacity; GSSG, oxidized glutathione; LP, lipid peroxidation; OSI, oxidative stress index; GR, glutathione reductase; LHP, lipid hydroperoxides; MDA, malondialdehyde; cyt-ox, cytochrome oxidase; ATA, atmosphere absolute; SCFAs, short-chain fatty acids.

References:

1. Ransberry, V. E., Blewett, T. A., McClelland, G. B. (2016). The oxidative stress response in freshwater-acclimated killifish (*Fundulus heteroclitus*) to acute copper and hypoxia exposure. *Comp. Biochem. Physiol. C Toxicol. Pharrmacol.* 179, 11-8. doi: 10.1016/j.cbpc.2015.08.001
2. Du, S. N., Mahalingam, S., Borowiec, B. G., Scott, G. R. (2016). Mitochondrial physiology and reactive oxygen species production are altered by hypoxia acclimation in killifish (*Fundulus heteroclitus*). *J. Exp. Biol.* 219, 1130-1138. doi: 10.1242/jeb.132860
3. Zhang, G., Mao, J., Liang, F., Chen, J., Zhao, C., Yin, S., et al. (2016). Modulated expression and enzymatic activities of Darkbarbel catfish, *Pelteobagrus vachelli* for oxidative stress induced by acute hypoxia and reoxygenation. *Chemosphere* 151, 271-279. doi: 10.1016/j.chemosphere.2016.02.072
4. Mustafa, S. A., Karieb, S. S., Davies, S. J., Jha, A. N. (2015). Assessment of oxidative damage to DNA, transcriptional expression of key genes, lipid peroxidation and histopathological changes in carp *Cyprinus carpio* L. following exposure to chronic hypoxic and subsequent recovery in normoxic conditions. *Mutagenesis* 30, 107-116. doi: 10.1093/mutage/geu048
5. Huang, C. Y., Lin, H. C., Lin, C. H. (2015). Effects of hypoxia on ionic regulation, glycogen utilization and antioxidative ability in the gills and liver of the aquatic air-breathing fish *Trichogaster microlepis*. *Comp. Biochem. Physiol. A Mol. Integr. Physiol.* 179, 25-34. doi: 10.1016/j.cbpa.2014.09.001
6. Leveelahti, L., Rytkönen, K. T., Renshaw, G. M., Nikinmaa, M. (2014). Revisiting redox-active antioxidant defenses in response to hypoxic challenge in both hypoxia-tolerant and hypoxia-sensitive fish species. *Fish Physiol. Biochem.* 40, 183-191. doi: 10.1007/s10695-013-9835-1
7. Hauser-Davis, R. A., Bastos, F. F., Dantas, R. F., Tobar, S. A., da Cunha Bastos Neto, J., da Cunha Bastos, V. L., et al. (2014). Behaviour of the oxidant scavenger metallothionein in hypoxia-induced neotropical fish. *Ecotoxicol. Environ. Saf.* 103, 24-28. doi: 10.1016/j.ecoenv.2014.01.015
8. Okogwu, O. I., Xie, P., Zhao, Y., Fan, H. (2014). Organ-dependent response in antioxidants, myoglobin and neuroglobin in goldfish (*Carassius auratus*) exposed to MC-RR under varying oxygen level. *Chemosphere* 112, 427-434. doi: 10.1016/j.chemosphere.2014.05.011
9. Dolci, G. S., Vey, L. T., Schuster, A. J., Roversi, K., Roversi, K., Dias, V. T., et al. (2014). Hypoxia acclimation protects against oxidative damage and changes in prolactin and somatolactin expression in silver catfish (*Rhamdia quelen*) exposed to manganese. *Aquat. Toxicol.* 157, 175-185. doi: 10.1016/j.aquatox.2014.10.015
10. Tripathi, R. K., Mohindra, V., Singh, A., Kumar, R., Mishra, R. M., Jena, J. K. (2013). Physiological responses to acute experimental hypoxia in the air-breathing Indian catfish, *Clarias batrachus* (Linnaeus, 1758). *J. Biosci.* 38, 373-383. doi: 10.1007/s12038-013-9304-0
11. Welker, A. F., Campos, E. G., Cardoso, L. A., Hermes-Lima, M. (2012). Role of catalase on the hypoxia/reoxygenation stress in the hypoxia-tolerant Nile tilapia. *Am. J. Physiol. Regul. Integr. Comp. Physiol.* 302, R1111-1118. doi: 10.1152/ajpregu.00243.2011
12. Pérez-Jiménez, A., Peres, H., Rubio, V. C., Oliva-Teles, A. (2012). The effect of hypoxia on intermediary metabolism and oxidative status in gilthead sea bream (*Sparus aurata*) fed on diets supplemented with methionine and white tea. *Comp. Biochem. Physiol. C Toxicol. Pharrmacol.* 155, 506-516. doi: 10.1016/j.cbpc.2011.12.005
13. Riffel, A. P., Garcia, L. O., Finamor, I. A., Saccol, E. M., Meira, M., Kolberg, C., et al. (2012). Redox profile in liver of *Leporinus macrocephalus* exposed to different dissolved oxygen levels. *Fish Physiol. Biochem.* 38, 797-805. doi: 10.1007/s10695-011-9563-3
14. Kubrak, O. I., Rovenko, B. M., Husak, V. V., Storey, J. M., Storey, K. B., Lushchak, V. I. (2012). Nickel induces hyperglycemia and glycogenolysis and affects the antioxidant system in liver and white muscle of goldfish *Carassius auratus* L. *Ecotoxicol. Environ. Saf.* 80, 231-237. doi: 10.1016/j.ecoenv.2012.03.006
15. Kelestemur, G. T. (2012). The antioxidant vitamin (A, C, E) and the lipid peroxidation levels in some tissues of juvenile rainbow trout (*Oncorhynchus mykiss*, W. 1792) at different oxygen levels. *Iran. J. Fish Sci.* 11, 315-324.
16. Azambujaa, C. R., Mattiazzia, J., Riffela, A. P. K., Finamora, I. A., Garciaa, L. D. O., Heldweinc, C. G., et al. (2011). Effect of the essential oil of Lippia alba on oxidative stress parameters in silver catfish (*Rhamdia quelen*) subjected to transport. *Aquaculture* 319, 156-161. doi: 10.1016/j.aquaculture.2011.06.002
17. Mustafa, S. A., Al-Subiai, S. N., Davies, S. J., Jha, A. N. (2011). Hypoxia-induced oxidative DNA damage links with higher level biological effects including specific growth rate in common carp, *Cyprinus carpio* L. *Ecotoxicology* 20, 1455-1466. doi: 10.1007/s10646-011-0702-5
18. Garcia Sampaio, F., de Lima Boijink, C., Tie Oba, E., Romagueira Bichara dos Santos, L., Lúcia Kalinin, A., Tadeu Rantin, F. (2008). Antioxidant defenses and biochemical changes in pacu (*Piaractus mesopotamicus*) in response to single and combined copper and hypoxia exposure. *Comp. Biochem. Physiol. C Toxicol. Pharrmacol.* 147, 43-51. doi: 10.1016/j.cbpc.2007.07.009
19. Lushchak, V. I., Bagnyukova, T. V. (2007). Hypoxia induces oxidative stress in tissues of a goby, the rotan *Perccottus glenii*. *Comp. Biochem. Physiol. B Biochem. Mol. Biol.* 148, 390-397. doi: 10.1016/j.cbpb.2007.07.007
20. Heise, K., Puntarulo, S., Nikinmaa, M., Lucassen, M., Pörtner, H. O., Abele, D. (2006). Oxidative stress and HIF-1 DNA binding during stressful cold exposure and recovery in the North Sea eelpout (*Zoarces viviparus*). *Comp. Biochem. Physiol. A Mol. Integr. Physiol.* 143, 494-503. doi: 10.1016/j.cbpa.2006.01.014
21. Olsvik, P. A., Kristensen, T., Waagbø, R., Tollefsen, K. E., Rosseland, B. O., Toften, H. (2006). Effects of hypo- and hyperoxia on transcription levels of five stress genes and the glutathione system in liver of Atlantic cod *Gadus morhua*. *J. Exp. Biol.* 209, 2893-2901. doi: 10.1242/jeb.02320
22. Lushchak, V. I., Bagnyukova, T. V., Lushchak, O. V., Storey, J. M., Storey, K. B. (2005). Hypoxia and recovery perturb free radical processes and antioxidant potential in common carp (*Cyprinus carpio*) tissues. *Int. J. Biochem. Cell Biol.* 37, 1319-1330. doi: 10.1016/j.biocel.2005.01.006
23. Wilhelm Filho, D., Torres, M. A., Zaniboni-Filho, E., Pedrosa, R. C. (2005). Effect of different oxygen tensions on weight gain, feed conversion, and antioxidant status in piapara, *Leporinus elongatus* (Valenciennes, 1847). *Aquaculture* 244, 349-357. doi: 10.1016/j.aquaculture.2004.11.024
24. Cooper, R. U., Clough, L. M., Farwell, M. A., West, T. L. (2002). Hypoxia-induced metabolic and antioxidant enzymatic activities in the estuarine fish *Leiostomus xanthurus*. *J. Exp. Mar. Biol. Ecol.* 279, 1-20. doi: 10.1016/S0022-0981(02)00329-5
25. Lushchak, V. I., Lushchak, L. P., Mota, A. A., Hermes-Lima, M. (2001). Oxidative stress and antioxidant defenses in goldfish *Carassius auratus* during anoxia and reoxygenation. *Am. J. Physiol. Regul. Integr. Comp. Physiol.* 280, 100-107
26. Lind, Y. (1992). Summertime and early autumn activity of some enzymes in the carbohydrate and fatty acid metabolism of the crucian carp. *Fish Physiol. Biochem.* 9, 409-415. doi: 10.1007/BF02274222
27. Radi, A. A., Matkovics, B., Csengeri, I. (1988). Effects of various oxygen concentrations on antioxidant enzymes and the quantity of tissue phospholipid fatty acids in the carp. *Acta Biol. Hung.* 39, 109-119.
28. Cann-Moisan, C., Sébert, P., Caroff, J., Barthélémy, L. (1988). Effects of hydrostatic pressure (HP = 101 ATA) on nucleotides and pyridine dinucleotides tissue contents in trout. *Exp. Biol.* 47, 239-242.
29. Arillo, A., Gaino, E., Margiocco, C., Mensi, P., Schenone, G. (1984) Biochemical and ultrastructural effects of nitrite in rainbow trout: liver hypoxia as the root of the acute toxicity mechanism. *Environ. Res.* 34, 135-154.
30. Burton, D. T., Spehar, A. M. (1971). A re-evaluation of the anaerobic endproducts of fresh-water fish exposed to environmental hypoxia. *Comp. Biochem. Physiol. A Comp. Physiol.* 40, 945-954. doi: 10.1016/0300-9629(71)90283-0
